# Introduction of Linda P. Fried, MD, MPH

**DOI:** 10.1172/JCI162514

**Published:** 2022-12-15

**Authors:** Anne B. Newman

**Affiliations:** 1 Department of Epidemiology, Center for Aging and Population Health, University of Pittsburgh, School of Public Health, Pittsburgh, Pennsylvania, USA.; 2 University of Pittsburgh, School of Medicine, Division of Geriatric Medicine, Pittsburgh, Pennsylvania, USA.

It is my honor and privilege to introduce my friend and colleague Dr. Linda P. Fried as the 2022 recipient of the Association of American Physicians (AAP) George M. Kober Medal. Dr. Fried is Dean of Columbia University’s Mailman School of Public Health, Professor of Epidemiology and DeLamar Professor of Public Health Practice at the Mailman School, Professor of Medicine at the Vagelos College of Physicians and Surgeons, and Senior Vice President of Columbia University Irving Medical Center.

Dr. Fried is also a past president of the AAP. In fact, I understand that she is only the second AAP president who is a dean of a school of public health, the first being William Henry Welch. In her AAP presidential address in 2017, she noted that one of the founders of the AAP was William Henry Welch. Welch started the Johns Hopkins School of Medicine, including the recruitment of the initial faculty, and served as founding dean from 1893 to 1898. He was president of AAP in 1901. He subsequently served as founding dean of the first school of public health, at Johns Hopkins, from 1916 to 1926. So here we are 100 years later, a second AAP president who is a public health school dean.

In researching Dr. Welch, I came to find that Dr. Welch was the first recipient of the Kober Medal (Figure 1) (1), bestowed by Dr. Kober himself in 1927 (Figure 2) (2).

Like Dr. Fried and Dr. Welch, George Kober was a strong advocate for public health. Throughout his career, he saw to it that preventive medicine and industrial hygiene were part of the medical school curriculum (3); in fact, his magnum opus was a textbook entitled *Industrial and Personal Hygiene* (4). I show here one of his early publications in *Science* where he defines the science of hygiene as the application of the teachings of physiology, chemistry, physics, meteorology — yes, meteorology — pathology, statistics, epidemiology, and bacteriology to the maintenance of the health and life of individuals and communities (2). This was published in 1897. I think that Dr. Kober would be quite pleased to see Dr. Linda Fried, with whom he shares a vision of public health as integral to medicine, receive this award in his name.

Let me tell you how I first came to know Dr. Fried. I first had the good fortune to meet her in 1988, at the first steering committee meeting of the Cardiovascular Health Study (CHS), a longitudinal cohort study of cardiovascular disease in older adults. Dr. Fried was the principal investigator for the Hopkins Field Center of the study. I must explain to you why I was so terribly impressed by her. First of all, she was the only woman leader in the study. I took note. She was also, at the time, a full generation younger than the other PIs, who were all men and quite senior. Furthermore, she was a geriatrician in a roomful of cardiologists and cardiovascular epidemiologists. I was so impressed by her leadership — she was always quite clear in her vision for the study, emphasizing what was important for older adults. At the same time, she was completely on top of all of the minute details of the protocol.

One point of discussion in the protocol was whether we should use the American Heart Association (AHA) classification of function for heart disease (I must note that this was a study funded by the National Heart, Lung, and Blood Institute). She explained to everyone why we needed to use new assessments of function that had been developed in the fields of geriatric medicine and epidemiology. She provided a sound scientific framework and cited data from previous studies. The steering committee soon came around to a decision to use the geriatric measures. It was unanimous. As a geriatrician myself, I was in perfect agreement, but when we left the room, I heard several people expressing concern that we were not following AHA dogma. Someone said, “Can you believe we are doing a cardiovascular study without the AHA classifications?!” In Linda’s inimitable manner, she just smiled and nodded and sailed on ahead. Here is a fairly recent photo of her CHS colleagues celebrating the study’s 25th anniversary, with best wishes from all of your CHS colleagues, Linda (Figure 3).

Some of her mentees have described Linda to me as a “force of nature.” Over the years, I have come to learn how several aspects of her unique background have led to her being such a force. First of all, you have to understand that Linda grew up in Manhattan. Her local library was the New York Public Library. Linda’s mother was a writer and a feminist who encouraged her daughter to think for herself. From an early age, she was led by her mother to believe that women could do anything they set their minds to. Add this to being a New Yorker and you have a recipe for accomplishment.

Another aspect of her background that I think is very important to understand was her early training in the martial art of aikido (Figure 4). She has often cited this training as essential to her ability to focus on the problem at hand and to maintain a sense of equilibrium. I had never thought of aikido as being useful for intellectual pursuits, but it may explain how Linda has maintained such focus and purpose in her work.

Linda’s choice to pursue medicine was an unusual path. Armed with a liberal arts degree and a black belt in aikido, she initially became a paralegal and then a social worker in the Department of Public Aid in Chicago. In the process, she came to understand that health is essential to social well-being and that social circumstances drive health outcomes. She pursued a degree in medicine from Rush Medical College, followed by internal medicine and public health training at Hopkins. She was convinced by Bill Hazzard (Figure 5) to add geriatric training and join the faculty in Geriatric Medicine at Hopkins, where she started one of the first geriatric assessment clinics.

Back in the 1980s, geriatric assessment clinics were in vogue to provide multidisciplinary assessments for frail older adults. Taking care of frailty was and continues to be the raison d’être of geriatric medicine (Figure 6). However, clinical trials of these geriatric assessment programs gave mixed results. Linda recognized that there was tremendous variability in targeting frailty and understood that this was a big part of the problem with these studies. For the most part, it was used almost colloquially as something that “you just know when you see it.” In fact, clinicians varied wildly in who was judged to be frail. Her view of frailty was grounded in a deep understanding of physiology. While developing a systems biology approach to defining frailty, she simultaneously sought to understand what mattered most to patients and their doctors. She used all of this to articulate a conceptual model of frailty, operationalized it with data, and validated it against meaningful clinical outcomes. In the Fried frailty model, frailty is the outward phenotypic expression of a state of vulnerability, characterized by signs and symptoms of a failure to maintain energetic homeostasis. These signs and symptoms include weakness, slowness, and fatigue, with weight loss and inactivity, a phenotype of a new clinical syndrome which she — and collaborators — have described (5) (Figure 7). Her groundbreaking publication on its validation has been cited over an astounding 18,000 times and continues to be cited frequently.

While continuing to pursue the science of frailty, Linda expanded her influence on the field as the Director of the Division of Geriatric Medicine and Gerontology and Founding Director of the Center on Aging and Health (COAH) at the Johns Hopkins Medical Institutions (JHMI). COAH is the institution’s Center of Excellence for aging research. Dr. Fried brought the Division of Geriatric Medicine and Gerontology to be ranked the no. one division in the US by *U.S*. *News and World Report* and brought research on aging, from basic to clinical to population science, at the JHMI to be highly transformed, integrated, and interdisciplinary sciences for health in aging. This has led the institution to be colloquially referred to as the Silicon Valley of Aging in the US. She procured an NIA program grant called a Pepper Center Grant (Figure 8) for Hopkins, which continues to be funded to this day.

She also established one of the first intervention research programs for prevention of frailty, a novel community-based program called the Experience Corps (Figure 9). This program provides supported volunteer roles for older adults to work with children in their neighborhood schools. The Experience Corps highlights Linda’s world view of aging, emphasizing the need for society to respond to the aging of the population with new opportunities for older adults to be active, productive, and generative. In the context of this important community program, she expanded frailty research by examining the physiologic responses to Experience Corp in a randomized clinical trial design. Armed with evidence of its benefit, she has continued to promote this important program nationwide and globally. It is now in 23 US cities and multiple countries.

Over more than two decades, Dr. Fried has continued to lead scientific investigation on frailty, demonstrating that there is a threshold level of dysregulation of multiple systems associated with the emergence of the frailty phenotype. There is now mounting evidence for these physiologic dysregulations being driven by alterations in the biologic hallmarks of aging, particularly dysfunction in mitochondria and energy production and intercellular communication as well as cellular senescence. The pathophysiology of frailty ultimately appears to be the consequence of a threshold level of disruption of the complex dynamical system of physiological resilience, as she described in the inaugural issue of *Nature Aging* (6). Today, frailty is understood to be a clinical syndrome and used clinically, diagnostically, and to determine which older adults can tolerate stressors such as surgery or chemotherapy and to design safer approaches for clinical management. With her collaborators, she and her mentees continue to explore frailty biology, most recently using novel assessments of resilience and pursuing novel therapies such as mesenchymal stem cell transplant.

With her capacity for scientific leadership and her vision for a healthy society, Linda expanded her influence in public health, becoming the Dean of the Mailman School of Public Health at Columbia in 2008. As dean, she has brought the science of aging and healthy longevity to new heights across Columbia, with the endowment of the University’s Butler Columbia Aging Center based in the Mailman School; she now serves as the Director. She has also built a range of innovative arenas of population health science for the 21st century, ranging from the first program on the Health Impacts of Climate Change in a school of public health, in 2009, to the programs in Precision Public Health, in Incarceration Prevention, and in Global Health Justice and Governance on a foundation of a life course approach to prevention and health promotion so as to extend health span to approximate our longer life expectancies. She has been active in promoting a response to COVID-19 that recognizes the social risks and consequences of the pandemic.

Many people talk about such remarkably accomplished leaders as role models. Dr. Fried has been a mentor for many and a role model for numerous women as well as men in medicine and public health. She trained scores of geriatrics and epidemiology fellows, doctoral students, and junior faculty who are now senior leaders in science, medicine, and public health in multiple institutions, including several members of AAP. Linda is a great role model, but in addition to having wonderful one-on-one relationships, she has also worked to transform institutions to address structural inequities for women in science. From 1989 to 1995, as an instructor and then Assistant Professor in Medicine, Dr. Fried served as the founding chair of the Johns Hopkins Department of Medicine’s Task Force on Women’s Academic Careers in Medicine. Appointed by John Stobo, MD, the Department Chair of Medicine, they jointly initiated and led a broad range of evidence-based interventions to establish equity of opportunity and success for women in Johns Hopkins Medicine and transformed the institutional culture and practices. The first round of results were published in 1996 in *JAMA* (7) along with multiple subsequent articles of analysis and evidence, and this initiative, sustained to the present, offered the first evidence that institutionally led interventions could create profound and sustained change towards creating gender equity for women. Dr. Fried subsequently chaired the 2000 to 2004 President’s Task Force on Women for the Johns Hopkins University, and her committee provided a similar roadmap for transformation university-wide to create gender equity for women faculty. She was also one of the first women in the Executive Leadership in Academic Medicine Program (ELAM) where she continues to serve as a mentor to many women in science and medicine.

In addition to these impressive professional roles, Linda is also a wife and mother of two sons. Her husband, Joe Margolick, the man to her left in the photo, is here with her today — with her sons, Alex and John, in virtual support (Figure 10).

Dr. Fried is the recipient of numerous honors and awards. Elected as a member of AAP in 2000, she served as president from 2016 to 2017. Elected as a member of the National Academy of Medicine (NAM) in 2000, she is in her second elected term as a member of NAM’s Executive Council. She is the recipient of the INSERM International Prize in Medical Research, the Alma Dea Morani Renaissance Woman Award from the Women in Medicine Legacy Foundation, and the Silver Scholar and Silver Innovator Awards from the Association for Aging Research, among many others (Figure 11). Dr. Fried was cited by Thomson Reuters in 2014 as being among the top 1% most influential scientific minds of the past decade and by the *New York Times* as among 15 top scientists. Most recently, she was honored by *Crain’s New York* as a 2020 Health Care Notable for leading her entire school in a strong response to bring science to strengthen the local, national, and global response to COVID-19.

In summary, Dr. Fried fully exemplifies all of the qualities embodied by the AAP in the Kober Medal. Please join me in welcoming her to come forward to receive this important distinction (Figure 12).

## Figures and Tables

**Figure 1:**
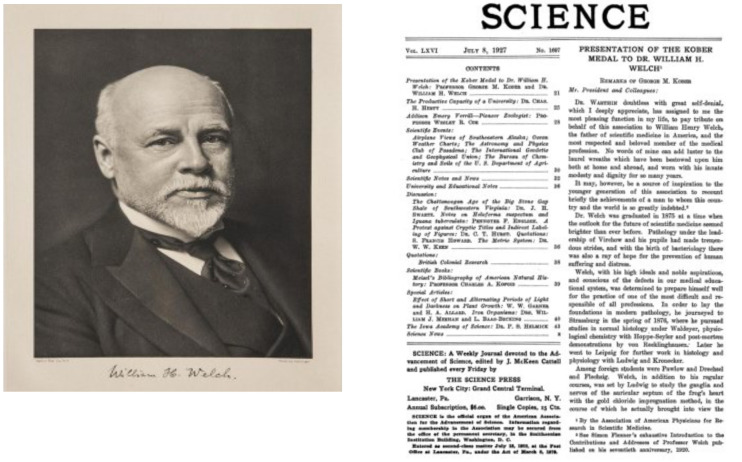
William Henry Welch. National Library of Medicine, public domain (1).

**Figure 2:**
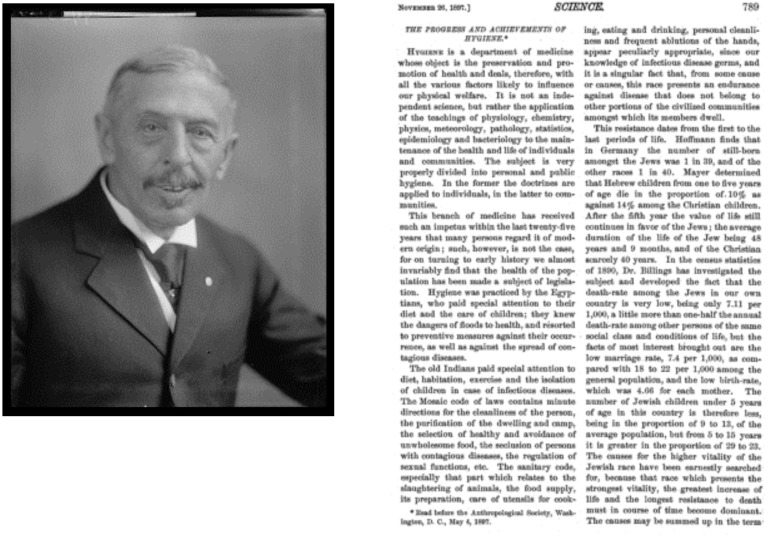
George M. Kober. National Library of Medicine, public domain (2).

**Figure 3:**
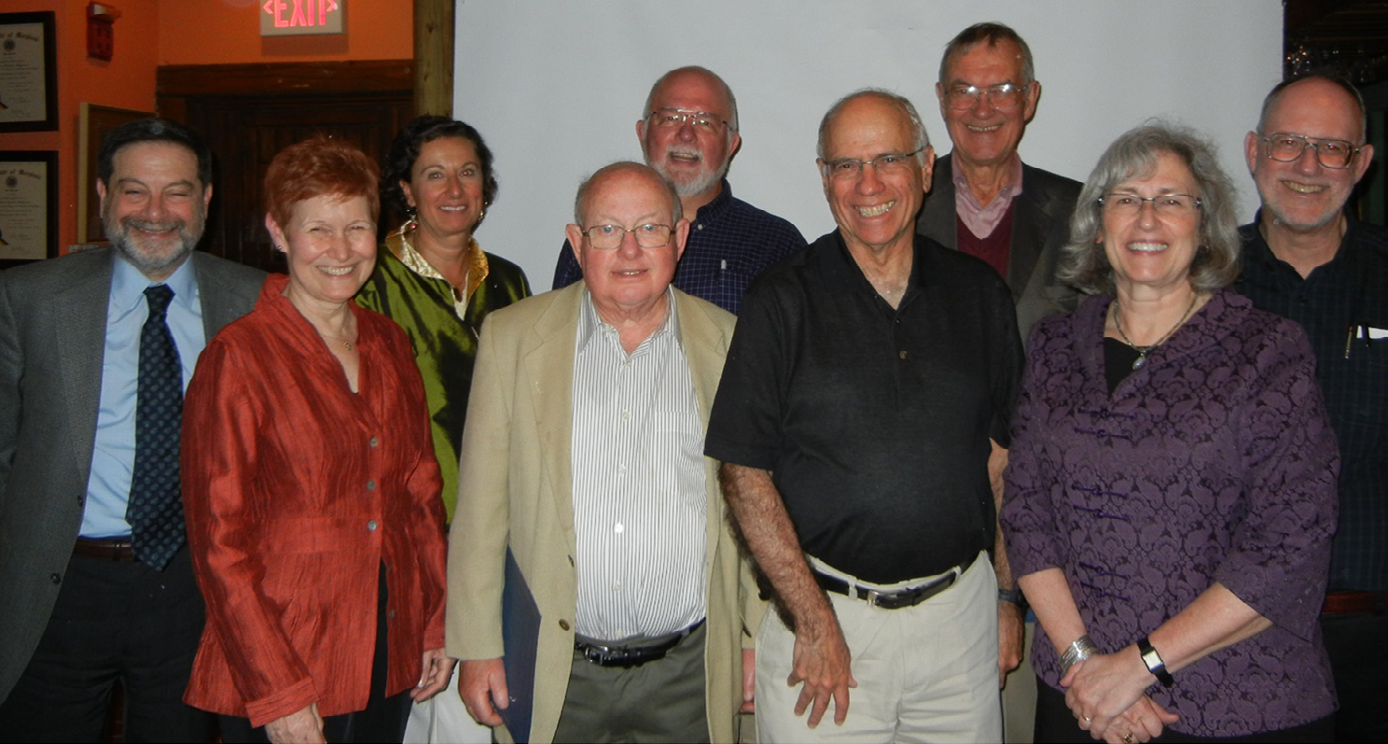
CHS colleagues. Left to right: David Siscovick, Pat Crowley, Diane Ives, Lew Kuller, Russ Tracy, Dick Kronmal, Curt Furberg, Anne Newman, and Bruce Psaty.

**Figure 4:**
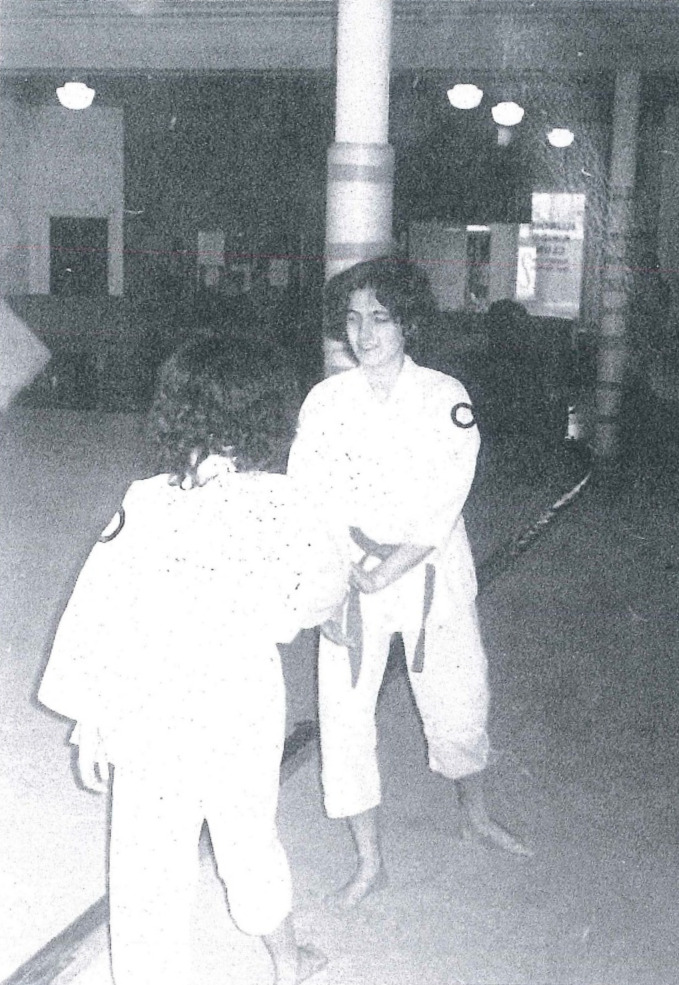
Dr. Linda Fried, practicing the martial art aikido.

**Figure 5:**
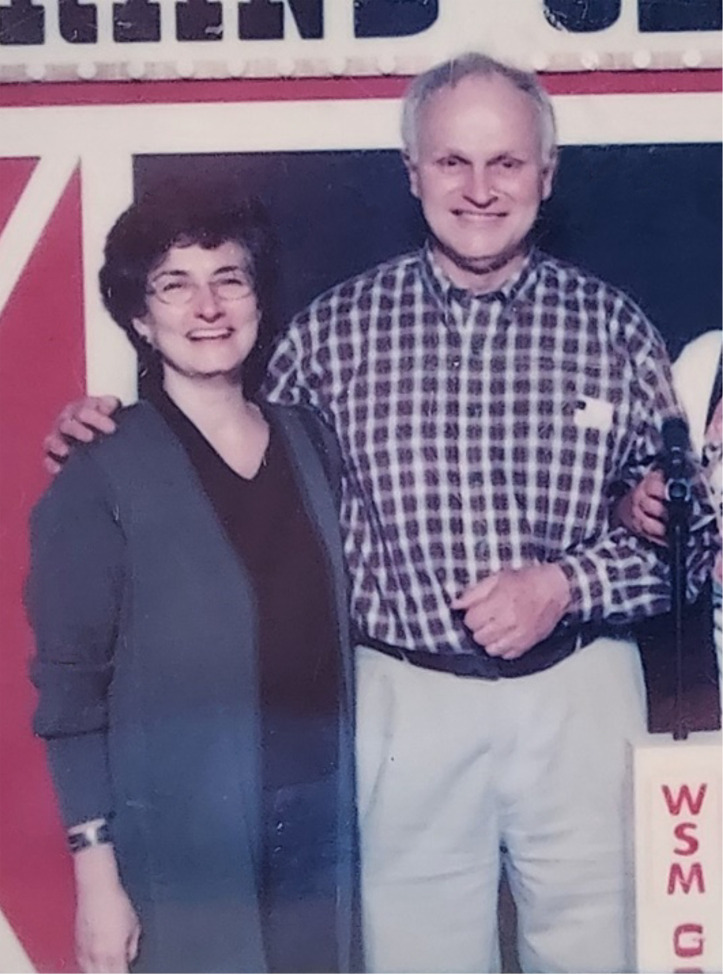
Linda Fried with Bill Hazzard.

**Figure 6:**
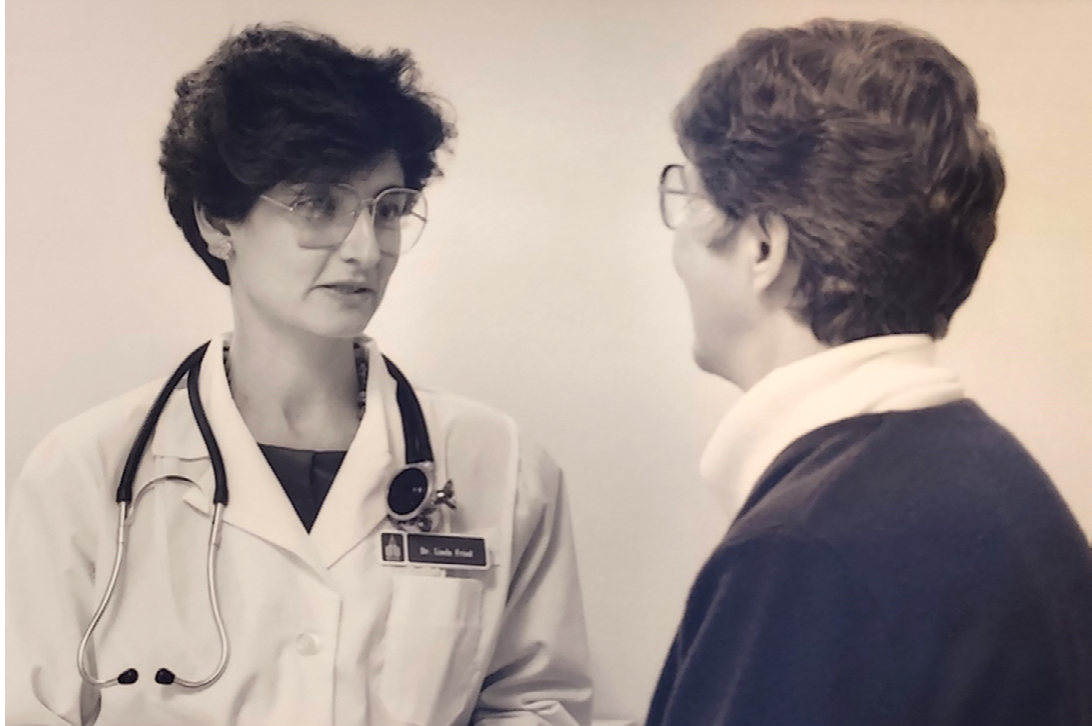
Dr. Linda Fried practicing geriatric medicine.

**Figure 7:**
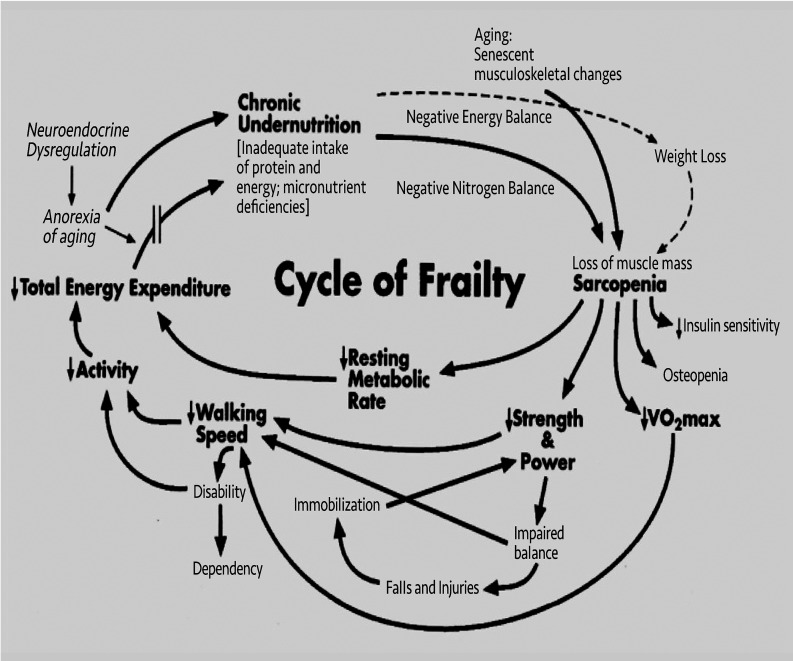
Frailty in older adults: evidence for a phenotype (5).

**Figure 8:**
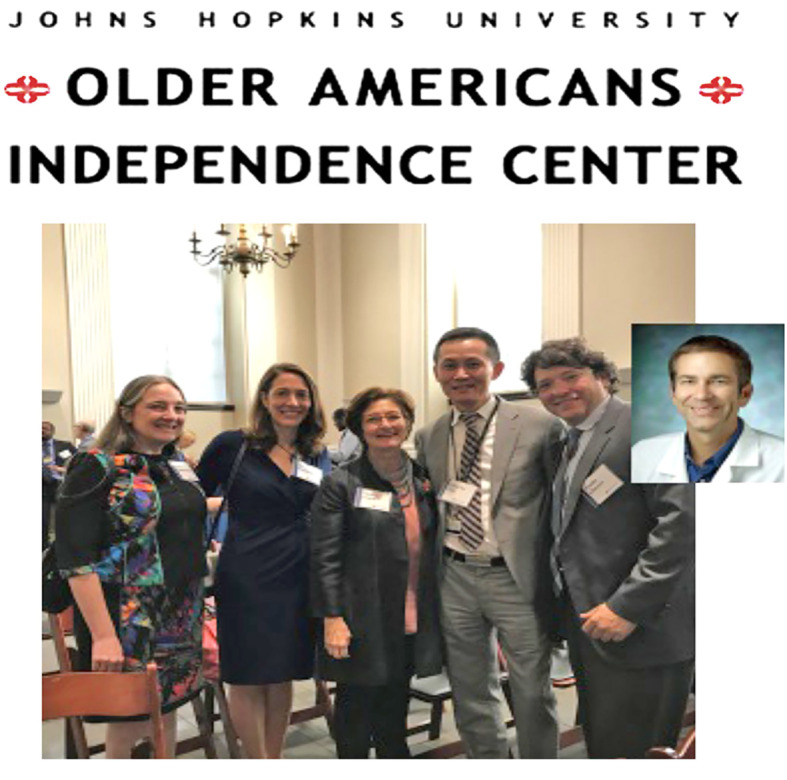
Johns Hopkins University Pepper Older Americans Independence Center.

**Figure 9:**
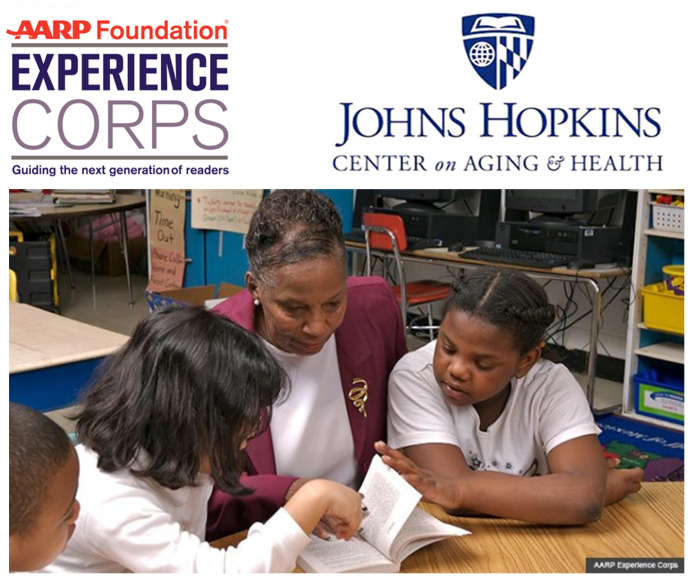
Experience Corps at Johns Hopkins University Center on Aging and Health.

**Figure 10:**
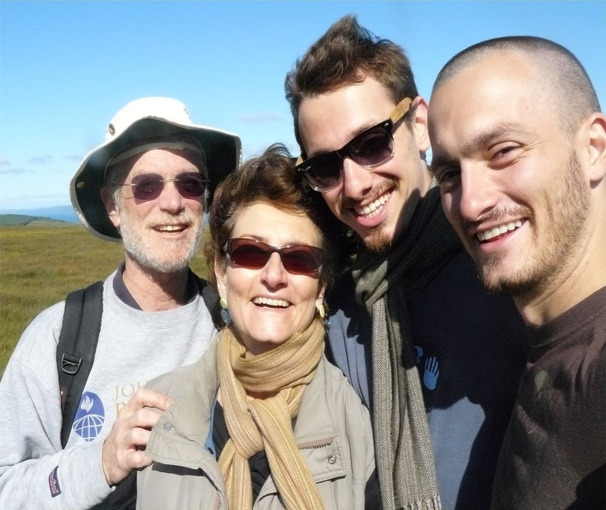
Dr. Linda Fried with her family: husband, Joe Margolick (left), and sons, Alex and John Margolick (right).

**Figure 11:**
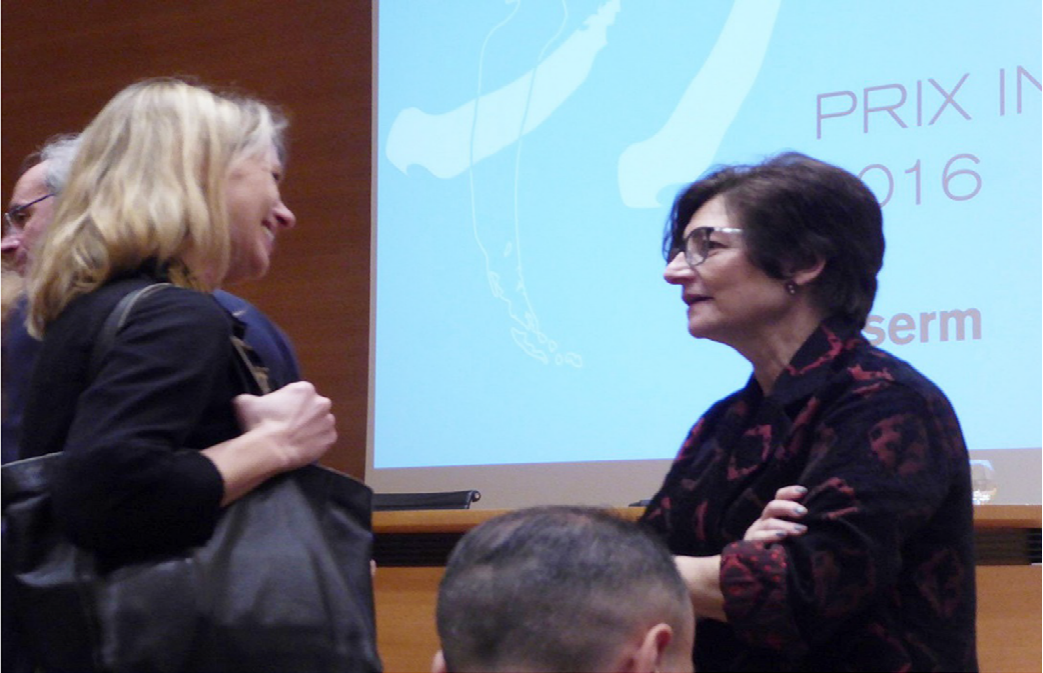
Dr. Fried receiving the INSERM International Prize in Medical Research.

**Figure 12:**
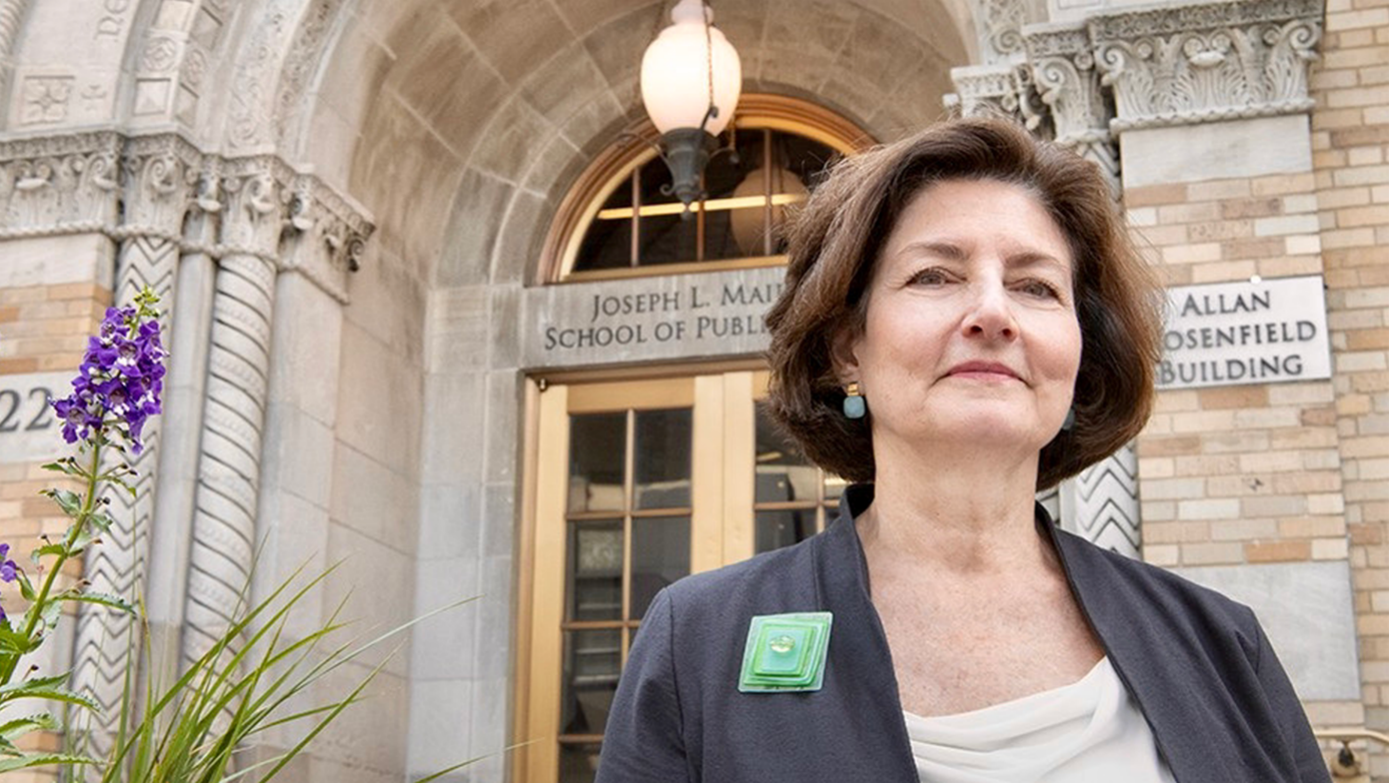
Linda P. Fried, MD, MPH, Dean of the Mailman School of Public Health and DeLamar Professor of Public Health Practice, Professor of Epidemiology and Medicine, Senior Vice President, Columbia University Medical Center.

## Version 1 12/15/2022

Electronic publication
